# Nutritional Status of Children with Eosinophilic Esophagitis: A Long-Term Follow-Up Study

**DOI:** 10.3390/nu18111710

**Published:** 2026-05-27

**Authors:** Marta Joanna Borys, Andrea Horvath, Piotr Dziechciarz

**Affiliations:** 1 Department of Paediatrics, Medical University of Warsaw, Żwirki i Wigury 63A, 02-091 Warsaw, Poland; martajoannaborys@gmail.com (M.J.B.); a.horvath@wum.edu.pl (A.H.); 2 Department of Methodology, Medical University of Warsaw, Żwirki i Wigury 61, 02-091 Warsaw, Poland

**Keywords:** eosinophilic esophagitis, malnutrition, obesity, growth, nutritional status, pediatric gastroenterology

## Abstract

Background/Objectives: To evaluate the long-term effects of eosinophilic esophagitis (EoE) on the nutritional status and growth of children. Methods: We performed a retrospective cohort study to assess longitudinal growth patterns (height and BMI z-scores) in pediatric patients (<18 years) newly diagnosed with EoE and followed for at least one year. Nutritional status was classified using BMI-based criteria from the Academy of Nutrition and Dietetics/American Society for Parenteral and Enteral Nutrition and the World Health Organization. Results: Among 50 patients, 20% presented with impaired nutritional status at diagnosis, including 12% with moderate malnutrition (BMI z-score < −2) and 8% with obesity (BMI z-score > +2). After a mean follow-up of 24.5 months, the prevalence of moderate malnutrition decreased to 6%, whereas obesity increased to 12%. Height z-scores remained largely stable over the follow-up period. Conclusions: EoE affects children across the full BMI spectrum. Long-term follow-up highlights the importance of monitoring nutritional status in all pediatric patients with EoE, given the risks of both malnutrition and obesity during disease management.

## 1. Introduction

Eosinophilic esophagitis (EoE) is a chronic immune-mediated esophageal disease characterized histologically by eosinophil-predominant inflammation of the esophagus and clinically by symptoms related to esophageal dysfunction [1]. EoE was first described as a distinct disease entity in the early 1990s. Since then, both the incidence and prevalence have increased substantially. Current estimates indicate an overall prevalence of approximately 34.4 per 100,000 individuals in Europe and North America [2]. In the pediatric population specifically, a population-based study from Utah reported an average annual incidence of 24 cases per 100,000 children and a prevalence of 118 cases per 100,000 children [3]. A systematic review with meta-analysis demonstrated a progressive increase in EoE prevalence over time, with pooled prevalence estimates rising approximately fourfold from early studies to those using current diagnostic criteria [4].

EoE exhibits a bimodal peak incidence between ages 5–10 years and in the fourth decade of life, with males affected 3–4 times more commonly than females [5]. EoE is currently considered the most frequent eosinophilic gastrointestinal disorder and the second most common cause of chronic esophagitis after gastroesophageal reflux disease [6].

The clinical presentation of EoE varies with age and includes food refusal, dysphagia, vomiting or regurgitation, upper abdominal or chest pain, food impaction, and failure to thrive. Published pediatric studies report failure to thrive or malnutrition in 10–30% of patients at diagnosis [3,7,8,9,10]. Several factors may negatively affect nutritional status in children with EoE, including recurrent vomiting, nausea, swallowing difficulties, gagging, and dysphagia. These factors may compromise feeding behavior, leading to feeding disorders that further impair nutritional status.

During therapy, patients may also experience altered nutritional status due to persistent maladaptive learned feeding behaviors [11]. Lack of response to therapy or poor treatment adherence may lead to sustained esophageal inflammation, which may additionally contribute to altered nutritional status [12]. Furthermore, prolonged elimination diet therapy may reduce adequate oral intake of food. Most studies evaluating nutritional status in pediatric EoE have focused only on the time of diagnosis, and there remains a paucity of long-term data [3,13]. Therefore, the aim of this study was to assess long-term changes in nutritional status and linear growth in a cohort of children diagnosed with EoE.

## 2. Materials and Methods

This retrospective follow-up study was conducted at the Pediatric Teaching Clinical Hospital of the Medical University of Warsaw, Poland. Patients were eligible if they met the following criteria: (1) confirmed diagnosis of EoE, (2) age < 18 years at diagnosis, and (3) follow-up duration of at least one year. Patients were excluded if anthropometric data at diagnosis or follow-up were unavailable.

The diagnosis of EoE was based on the International Consensus Diagnostic Criteria for Eosinophilic Esophagitis [1]. Diagnostic criteria included symptoms of esophageal dysfunction, histopathological findings of at least 15 eosinophils per high-power field (equivalent to 60 eosinophils/mm^2^), and exclusion of other causes of esophageal eosinophilia.

Clinical data were extracted from electronic medical records. Anthropometric measurements were performed by trained personnel using calibrated stadiometers and medical scales. Weight and height measurements were obtained to the nearest 0.1 kg and 0.1 cm, respectively. Although measurements were not performed by the same examiner at every visit, standardized WHO-recommended measurement protocols were followed.

Body mass index (BMI) was calculated as weight divided by height squared (kg/m^2^). Age-standardized z-scores for height and BMI were calculated using WHO Anthro and WHO AnthroPlus software [14,15].

Patients were evaluated at diagnosis and at the final follow-up visit for the following outcomes:

- Moderate malnutrition, defined as BMI z-scores < −2
- Obesity, defined as BMI z-scores > +2
- Short stature, defined as height z-scores < −2
- Tall stature, defined as height z-scores > +2

Definitions were based on recommendations from the Academy of Nutrition and Dietetics/American Society for Parenteral and Enteral Nutrition (ADA/ASPEN), WHO criteria, and the European Society for Pediatric Endocrinology classification system [16].

To assess longitudinal changes in anthropometric parameters, paired-samples *t*-tests were performed for BMI z-scores and height z-scores. The normality assumptions were considered acceptable given the sample size. As a sensitivity analysis for BMI and height, a non-parametric Wilcoxon signed-rank test was additionally performed. A *p*-value < 0.05 was considered statistically significant. All statistical analyses were conducted using Statistica (StatSoft, Tulsa, OK, USA).

Additional baseline information included sex, age at diagnosis, concomitant diseases, and Index of Severity for Eosinophilic Esophagitis (I-SEE) scores. Follow-up data included clinical remission assessed by treating physician, I-SEE scores, and histological remission defined as <15 eosinophils per high-power field across two levels of the esophagus.

Endoscopic evaluation with biopsies was performed according to clinical indication and treatment response, typically 8–12 weeks after initiation or modification of therapy, consistent with guideline recommendations [1,17]. Histological remission data represented the most recent available biopsy results for each patient.

All data were collected using a predefined standardized data extraction protocol. The study was approved by the Ethics Committee of the Medical University of Warsaw (protocol number 1701/2023). Descriptive statistics were used to describe the study population and outcomes. Data are presented as numbers and percentages or means with 95% confidence intervals (95% CI), as appropriate.

## 3. Results

Initially, 58 patients diagnosed with EoE between July 2017 and July 2019 were identified. Eight patients were excluded due to inconclusive histopathology (n = 1), loss to follow-up (n = 2), discontinuation of treatment (n = 1), or missing anthropometric data (n = 4). Consequently, 50 patients fulfilled the inclusion criteria (Table 1).

**Figure 1:**
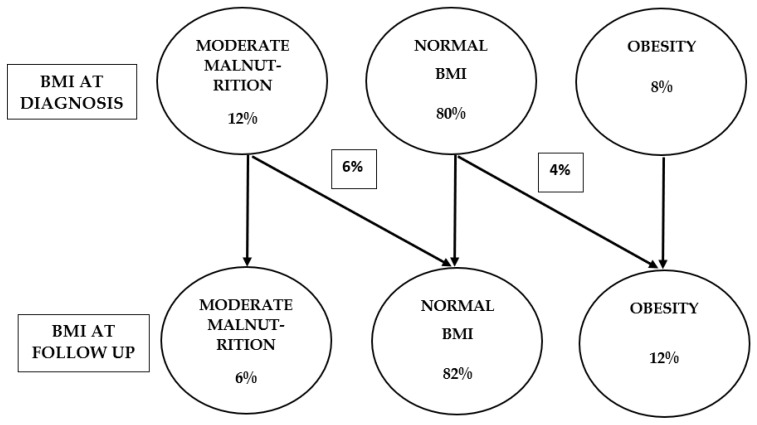
Distribution to BMI groups at diagnosis of eosinophilic esophagitis and follow-up.

The mean treatment duration was 24.5 months (range: 12.5–35 months), with a median of 23 months. At diagnosis, the mean BMI z-score was 0.002 (95% CI: −0.72 to 0.73). Moderate malnutrition was present in 8% of patients, while an additional 4% had severe malnutrition (BMI z-score < −3), resulting in 12% classified as malnourished overall. Obesity was identified in 8% of children (Figure 1).

At the end of follow-up, the mean BMI z-score increased slightly to 0.025 (95% CI: −0.63 to 0.81). Six percent of patients had moderate malnutrition, whereas obesity prevalence increased to 12% (Figure 1).

A paired-samples *t*-test showed a non-significant increase in BMI z-scores from −0.02 at baseline to 0.15 at follow-up, corresponding to a mean change of 0.17 (t(50) = 1.47, *p* = 0.147). The 95% confidence interval for the mean difference ranged from −0.06 to 0.40. Consistent results were obtained using a Wilcoxon signed-rank test, which also indicated no statistically significant change (*W* = 548.0, *p* = 0.388).

The mean height z-score at diagnosis was 0.12 (95% CI: −0.65 to 0.89) and increased slightly to 0.24 (95% CI: −0.35 to 1.03) at follow-up.

A paired-samples *t*-test demonstrated no statistically significant difference in height z-scores between baseline (mean 0.44) and follow-up (mean 0.46), with a mean change of 0.01 (t(49) = 0.12, *p* = 0.907). The 95% confidence interval for the mean difference ranged from −0.19 to 0.21.

Overall, neither BMI nor height z-scores changed significantly over time, indicating relative stability of nutritional status and linear growth during the follow-up period.

**Figure 2:**
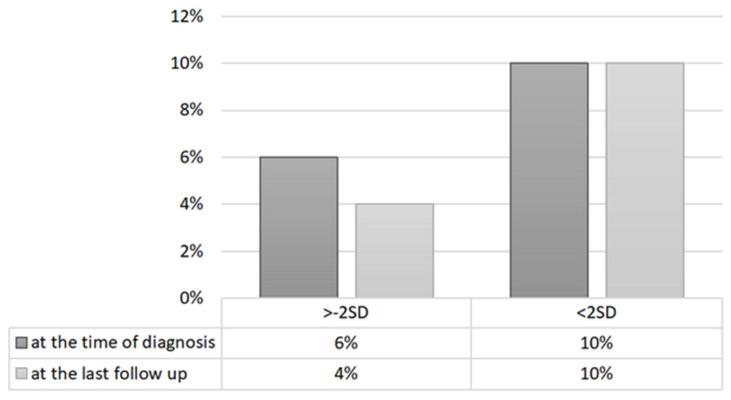
Proportion of children with tall and short stature at the time of diagnosis and at the last visit.

The mean height z-score at diagnosis was 0.12 (95% CI: −0.65 to 0.89). At the end of the follow-up period, the mean height z-score increased slightly to 0.24 (95% CI: −0.35 to 1.03). Short stature was present in 4% of patients at diagnosis and remained unchanged at follow-up. Tall stature was observed in 10% of patients throughout the study period (Figure 2). A paired-samples *t*-test demonstrated no statistically significant difference in height z-scores between baseline (mean 0.44) and follow-up (mean 0.46), with a mean change of 0.01 (t(50) = 0.12, *p* = 0.907). The 95% confidence interval for the mean difference ranged from −0.19 to 0.21. Consistent results were obtained using a non-parametric approach; the Wilcoxon signed-rank test also showed no statistically significant change in height z-scores (*W* = 480.5, *p* = 0.676).

At diagnosis, all but one patient (98%) initiated therapy with proton pump inhibitors. During follow-up, 78% received some form of elimination diet, including 30% treated with an elemental diet. Regarding topical corticosteroid exposure, 56% of patients received topical steroids during follow-up. However, only two patients (4%) received continuous topical steroid therapy for longer than three months.

At the final follow-up visit, 86% of patients were considered to be in clinical remission as assessed by their treating physicians, whereas 56% achieved histological remission. Nevertheless, 32% of patients still demonstrated severe active EoE, and 8% demonstrated moderately active disease according to I-SEE scores (Table 2).

## 4. Discussion

### 4.1. Summary of the Results

This study evaluated long-term nutritional status and growth among pediatric patients diagnosed with EoE. At diagnosis, 20% of children demonstrated impaired nutritional status according to BMI criteria, including 12% with malnutrition and 8% with obesity. Following a mean follow-up duration of 24.5 months, the prevalence of malnutrition decreased to 6%, whereas obesity prevalence increased modestly to 12%. Linear growth remained largely preserved throughout follow-up, with stable mean height z-scores and a low prevalence of short stature.

These findings emphasize that EoE may affect children across the full spectrum of nutritional status. Although EoE has traditionally been associated with failure to thrive and undernutrition, our findings highlight that obesity may also occur in pediatric EoE. This observation supports the need for clinicians to consider EoE regardless of baseline BMI status.

The study also underscores the importance of long-term nutritional monitoring in pediatric EoE. Nutritional follow-up should not focus exclusively on undernutrition but should additionally address excessive weight gain and obesity. Nutritional counseling should therefore be individualized and adapted to the patient’s nutritional status, disease activity, dietary restrictions, and feeding behaviors.

Several mechanisms may contribute to undernutrition in pediatric EoE. Recurrent vomiting, dysphagia, food refusal, prolonged elimination diets, and maladaptive feeding behaviors may substantially reduce oral intake [11,18]. Persistent feeding dysfunction may continue even after histological remission due to learned eating avoidance behaviors and esophageal remodeling [19]. Conversely, obesity may result from increased intake of calorie-dense “safe” foods, reduced physical activity associated with chronic disease, or improvement in dysphagia leading to increased caloric intake without corresponding dietary counseling.

Importantly, linear growth remained largely unaffected in our cohort. These findings are consistent with previous studies demonstrating that growth impairment secondary to swallowed topical corticosteroids is relatively uncommon in EoE [12]. The limited duration of prolonged steroid exposure in our cohort likely contributed to the preservation of normal linear growth.

### 4.2. Clinical Versus Histological Discrepancies

One of the most clinically relevant findings of the present study was the discordance between high clinical remission rates and persistent disease activity according to I-SEE scores. Although 86% of patients were in clinical remission at follow-up, only 56% achieved histological remission, and approximately one-third still demonstrated severe active disease according to I-SEE criteria.

This symptom–histology dissociation reflects an increasingly recognized characteristic feature of EoE [1,17]. Several mechanisms may explain this discrepancy.

Adaptive eating behaviors appear to be among the most important contributors. Patients with EoE frequently develop compensatory feeding strategies, including prolonged chewing, avoidance of certain food textures, smaller bite sizes, and increased liquid intake during meals [19,20]. These behaviors may reduce symptom perception despite persistent esophageal inflammation. Importantly, adaptive eating patterns may persist even after histological improvement, driven by esophageal remodeling and learned feeding avoidance.

Disease-related anxiety and hypervigilance may additionally influence symptom perception. Recent studies suggest that many patients with EoE develop esophageal-specific anxiety, leading to altered eating behaviors and symptom reporting [20]. Such psychological adaptations may complicate clinical assessment and contribute to underestimating ongoing disease activity.

Another contributing factor may be esophageal remodeling and fibrosis. Chronic inflammation may progressively decrease esophageal distensibility, leading patients to unconsciously adapt their eating patterns over time [19]. Consequently, symptoms may not accurately reflect inflammatory activity.

Importantly, the I-SEE score incorporates multiple domains beyond clinical symptoms, including endoscopic and histological features. Therefore, an increase in severe disease according to I-SEE may occur despite apparent clinical improvement.

Consequently, clinical remission should not be interpreted as a surrogate marker of complete disease control.

### 4.3. Comparison with Other Studies

Previous pediatric studies have reported growth impairment or malnutrition in approximately 10–30% of children with EoE [3,7,8,9,10]. Differences between studies may partly reflect variations in malnutrition definitions, study populations, referral patterns, and treatment strategies.

Compared with prior reports, the prevalence of malnutrition in our cohort was relatively low. This may be explained by our use of conservative ADA/ASPEN criteria, which classified only moderate and severe malnutrition as clinically significant. Many earlier studies used broader definitions of failure to thrive, potentially resulting in higher prevalence estimates.

Interestingly, obesity in pediatric EoE remains relatively understudied. Most earlier reports focused primarily on undernutrition and growth failure. In our cohort, obesity affected 8% of children at diagnosis and 12% during follow-up. These findings are consistent with previous observations from Poland demonstrating that obesity may occur in children with EoE [13]. The increasing prevalence of obesity observed during follow-up may reflect broader epidemiological trends in pediatric obesity as well as disease-specific dietary adaptations.

Only a limited number of studies have assessed long-term nutritional outcomes in pediatric EoE using BMI z-scores [3,12]. Most available studies have focused on shorter follow-up periods and changes in mean anthropometric parameters rather than detailed nutritional categories. Therefore, our study contributes additional long-term data regarding both undernutrition and obesity in pediatric EoE.

Two patients in our cohort had concomitant celiac disease. Although celiac disease may independently influence nutritional status and growth, previous studies suggest that EoE and celiac disease likely represent coexisting disorders rather than manifestations of a single disease process [18]. Due to the small number of affected patients, subgroup analyses were not feasible.

### 4.4. Strengths and Limitations

The present study has several strengths. First, the relatively long follow-up duration distinguishes this study from many previous pediatric EoE studies. Second, we used predefined and standardized anthropometric definitions based on internationally recognized criteria. Third, anthropometric assessments were performed by trained personnel using calibrated equipment and standardized protocols. Finally, the study included a detailed longitudinal evaluation of nutritional status, growth, and disease activity.

Nevertheless, several limitations should be acknowledged. The retrospective design required reliance on medical record abstraction and may therefore be subject to incomplete data collection or documentation bias. Anthropometric measurements were not performed by a single examiner, although all personnel followed standardized procedures.

The study was additionally limited by its single-center design and relatively modest sample size, which may reduce generalizability. The sample size also precluded meaningful subgroup analyses according to treatment modality. Such analyses were further complicated by the dynamic nature of EoE treatment, as many patients changed therapies during follow-up. Most patients initially received proton pump inhibitors before transitioning to elimination diets or topical corticosteroids.

Another important limitation is the lack of detailed nutritional data. Information regarding dietary intake, adherence to elimination diets, use of dietary supplements, and involvement of dietitians was not systematically available. These factors may significantly influence nutritional outcomes and represent an important area for future research.

Finally, histological follow-up timing was not fully standardized, as endoscopic reassessment was guided by clinical indications and treatment response. However, this approach reflects real-world clinical practice.

## 5. Conclusions

EoE can affect children across the entire spectrum of nutritional status, ranging from malnutrition to obesity. Long-term monitoring of nutritional status and growth should therefore be incorporated into the routine management of pediatric EoE. Clinicians should remain aware that both undernutrition and obesity may develop during the disease course. Personalized nutritional counseling and careful longitudinal follow-up are essential to optimize growth and nutritional outcomes in children with EoE.

## Figures and Tables

**Table 1:** Characteristics of study participants.

Characteristic	Value
Gender	
- Male	42 (84)
- Female	8 (16%)
Age at diagnosis	
- median	12.4 years
- range	1–16 years
Concomitant diseases:	
- Asthma	6
- Allergic rhinitis	4
- Pollen food allergy syndrome	1
- Celiac disease	2
- H.pylori gastritis	7
- Depression	1

**Table 2:** Index of Severity for Eosinophilic Esophagitis, assessed using available data at diagnosis and at the last visit.

Severity Level	At Diagnosis n (%)	At the Last Visit n (%)
inactive	0 (0%)	2 (4%)
mild active	32 (64%)	28 (56%)
moderate active	8 (16%)	4 (8%)
severe	10 (20%)	16 (32%)

## Data Availability

Data are available from the corresponding author upon reasonable request.
